# Prevalence of Newcastle disease and associated risk factors in domestic chickens in the Indian state of Odisha

**DOI:** 10.1371/journal.pone.0264028

**Published:** 2022-02-16

**Authors:** Niranjana Sahoo, Kashyap Bhuyan, Biswaranjan Panda, Nrushingha Charan Behura, Sangram Biswal, Lipismita Samal, Deepika Chaudhary, Nitish Bansal, Renu Singh, Vinay G. Joshi, Naresh Jindal, Nand K. Mahajan, Sushila Maan, Chintu Ravishankar, Ravindran Rajasekhar, Jessica Radzio-Basu, Catherine M. Herzog, Vivek Kapur, Sunil K. Mor, Sagar M. Goyal

**Affiliations:** 1 Department of Epidemiology & Preventive Medicine and Poultry Science, College of Veterinary Science & Animal Husbandry, Odisha University of Agriculture and Technology, Bhubaneswar, Odisha, India; 2 Departments of Veterinary Public Health & Epidemiology and Animal Biotechnology, College of Veterinary Sciences, LalaLajpat Rai University of Veterinary and Animal Sciences, Hisar, Haryana, India; 3 Department of Veterinary Microbiology, College of Veterinary and Animal Sciences, Kerala Veterinary and Animal Sciences University, Pookode, Kerala, India; 4 The Huck Institute of the Life Sciences, The Pennsylvania State University, University Park, Pennsylvania, United States of America; 5 Department of Animal Science, The Pennsylvania State University, University Park, Pennsylvania, United States of America; 6 Department of Veterinary Population Medicine, University of Minnesota, St. Paul, Minnesota, United States of America; University of Connecticut, UNITED STATES

## Abstract

Newcastle disease (ND), caused by Newcastle disease virus (NDV), is a contagious disease that affects a variety of domestic and wild avian species. Though ND is vaccine-preventable, it is a persistent threat to poultry industry across the globe. The disease represents a leading cause of morbidity and mortality in chickens. To better understand the epidemiology of NDV among commercial and backyard chickens of Odisha, where chicken farming is being prioritized to assist with poverty alleviation, a cross-sectional study was conducted in two distinct seasons during 2018. Choanal swabs (*n* = 1361) from live birds (commercial layers, broilers, and backyard chicken) and tracheal tissues from dead birds (*n* = 10) were collected and tested by real-time reverse transcription polymerase chain reaction (RT-PCR) for the presence of matrix (M) and fusion (F) genes of NDV. Risk factors at the flock and individual bird levels (health status, ND vaccination status, geographical zone, management system, and housing) were assessed using multivariable logistic regression analyses. Of the 1371 samples tested, 160 were positive for M gene amplification indicating an overall apparent prevalence of 11.7% (95% CI 10.1–13.5%). Circulation of virulent NDV strains was also evident with apparent prevalence of 8.1% (13/160; 95% CI: 4.8–13.4%). In addition, commercial birds had significantly higher odds (75%) of being infected with NDV as compared to backyard poultry (*p* = 0.01). This study helps fill a knowledge gap in the prevalence and distribution of NDV in apparently healthy birds in eastern India, and provides a framework for future longitudinal research of NDV risk and mitigation in targeted geographies—a step forward for effective control of ND in Odisha.

## Introduction

Newcastle disease (ND) is a well-known viral disease that poses a threat to the poultry industry globally even a century after its first description in 1926 in Indonesia. The Newcastle disease virus (NDV), the causative agent of ND, is classified under the family *Paramyxoviridae*, subfamily *Avulavirinae*, genus *Orthoavulavirus* and species *Avian orthoavulavirus* [1,2]. The genome of NDV is a single-stranded, negative-sense RNA, which is about 15kb in length. It encodes six genes in the order: nucleoprotein (NP), phosphoprotein (P), fusion protein (F), matrix protein (M), hemagglutinin-neuraminidase (HN), and RNA large polymerase (L) [3]. Based on pathogenicity, NDV is categorized into five pathotypes that include asymptomatic enteric, lentogenic, mesogenic, viscerotropic-velogenic, and neurotropic-velogenic strains. The lentogenic strains are widespread globally. However, velogenic strains are responsible for disease outbreaks in poultry. The amino acid sequence of the unprocessed F protein cleavage site determines the pathogenic potential of NDV in chickens [4].

The virus has a wide host range among avian species; at least 250 species classified in 27 of the 50 orders of birds are susceptible to NDV. However, its impact is most visible in domestic poultry in which NDV infection leads to major losses in productivity. The transmission of NDV is primarily through aerosol or oral routes. Based on the available literature, NDV ecology can be divided into two host systems: wild waterfowl harboring lentogenic strains [5] and domestic poultry in which outbreaks occur due to mesogenic/velogenic strains [6]. The highly pathogenic strains seem to arise during replication of the virus in high-density poultry populations [4,7].

Though vaccination with live or oil emulsion vaccines were developed decades ago and have reduced the morbidity and mortality-related losses, repeated ND outbreaks in chickens are reported even in vaccinated populations across the globe including North and Central America, Europe, Asia, the Middle East, and Africa [8]. In India, ND is considered as endemic [9] and is commonly known as the Ranikhet Disease (RD) among poultry farmers and veterinarians.

Odisha, an eastern state of India, is the eighth largest state in India with four million layers, which comprise approximately 2.5% of India’s total layer population. Presently, there are 8.5 million broilers, which are distributed in 5000 commercial broiler farms with capacities ranging from 200 to 15000 birds. In addition, there are 19.8 million backyard chickens reared under traditional rural management system, which comprises approximately 60% of the state’s total poultry population [10]. Additionally, Odisha has an internationally renowned natural wetland called the Chilika Lake, which is visited each winter (November- February) by more than one million migratory birds. The presence of commercial and backyard poultry farms near the vicinity of water bodies at different geolocations including Chilika Lake and along migratory birds’ fly-paths may create an opportunity for exchange of NDV among these bird populations. Resident water birds roaming year-round may also act as mediators of infection from commercial and backyard poultry to migratory birds and vice versa.

Due to the lack of routine surveillance programs in India, there is a considerable knowledge gap on circulating NDV strains in apparently healthy birds, and epidemiological features associated with ND outbreaks. To help fill this gap, a cross-sectional survey was conducted in 2018 to better understand NDV prevalence and associated risk factors in commercial and backyard poultry in Odisha.

## Materials and methods

### Source of birds

**Fig 1:**
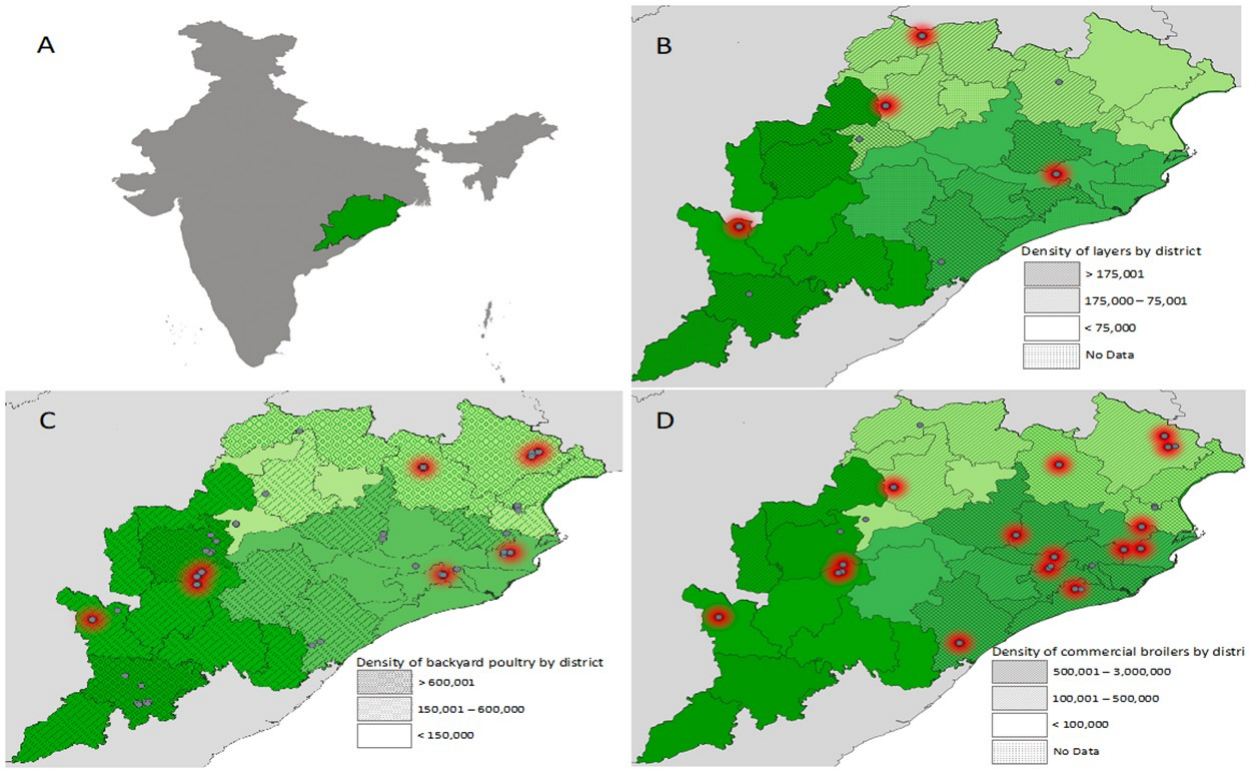
Distribution of M-gene positive samples across Odisha in sampling zones 1 (light greed), 2 (green) and 3 (dark green). The maps show the distribution of samples in Odisha (A) which tested positive (red, red halo) or negative (gray) for the NDV M-gene in layers (B), backyard birds (C) and commercial broilers (D). (*Map created using the Free and Open Source QGIS)*.

The state Odisha is conventionally divided into three physiographic zones (zone I- middle and northern mountainous highlands and central plateaus, zone II- eastern flood and coastal plains, and zone III- southern and western forest cover). Fig 1 depicts the density and distribution of commercial broilers, commercial layers and backyard poultry in these three zones of Odisha based on 19^th^ Livestock Census-2012 [10]. Three different types of birds (commercial broilers, layers, and backyard poultry) reared in these three zones were sampled during two seasons in 2018: migratory (November-February) and non-migratory (April-August). Stratified sampling approach was undertaken based on a power of 0.8 (probability of finding an effect that is there, Type II error), a significance level of 0.05 (probability of finding an effect that is not there, Type I error) and an effect size of 0.2 (i.e., level of difference between samples, 0.1 = low difference 0.8 = high difference). At the time of the farm visit, samples were collected from apparently healthy, dead and ill birds. Birds that were either dead or ill having signs other than respiratory signs were excluded from the study. Farms were categorized into three types namely small, medium and large. In backyard poultry farms, 4–5, 10–12 and 15–25 samples were collected from small, medium and large farms, respectively. In commercial broiler chicken farms, the sampling sizes were 10–15, 15–20 and 20–40 in small, medium and large categories, respectively. In case of commercial layers, 20–30 samples were collected from each farm except those with a population of 100,000 or more. In the latter category, the farm complex had several different sheds from which 30–35 samples were collected. Management systems were categorized as extensive, semi-intensive, and intensive on the basis of flock sizes of 1–20, 21–200, and >200 birds, respectively. This classification was based on a prior survey of the study area for other health related activities. Contrary to backyard poultry, vaccination against ND is a regular practice in commercial birds. The consent of individual poultry farmers was obtained after they were apprised of the purpose and benefits of this study.

### Sampling procedure and sample processing

Choanal swabs were collected during the study period by stratified sampling technique that covered the entire state. The samples were primarily collected from apparently healthy flocks, using sterile polyester swabs. Tracheal tissues were collected from dead birds, which had a history of clinical signs suggestive of NDV infection e.g., drowsiness, ocular nasal discharge, respiratory sounds and greenish droppings. Live birds showing clinical signs of ocular nasal discharge, drowsiness, swelling of face and eye, and white droppings were considered as ND suspect (sick). Samples were placed in 2.2 ml of Brain Heart Infusion (BHI) broth contained in pre-labeled 4.5mL cryovials. The samples were kept on ice after collection, brought to the laboratory, and centrifuged at 1200 x*g*. The supernatants were aliquoted and stored at -80°C until further processing. A 10% suspension of tissues was prepared in BHI broth followed by centrifugation at 122 xg for 20 min. Survey information on flock size, ND vaccination status, respiratory signs, disease history, and mortality were collected and recorded in a predesigned EpiCollect5 form [11, S1 Table].

### Detection of M and F genes of NDV

RNA was extracted from samples using ThermoFisher MagMAX^™^-96 viral RNA isolation kit following the kit manufacturer’s protocol. The NDV matrix gene (M gene) detection by real-time RT-PCR was carried out using Qiagen OneStep RT-PCR kit (Qiagen, Germany) in Stratagene MX3000P Real Time PCR machine (Stratagene, USA) by following protocol as reported previously [12,13]. In brief, the steps involved were reverse transcription at 50°C for 25 min, initial PCR activation at 95°C for 10 min, 40 cycles of denaturation at 95°C for 10 sec, and annealing and extension at 60°C for 30s. The total volume of the reaction was 12.5μL with 10pmol of both forward and reverse primer along with 5pmol of probe (Table 1). The efficiency of the M gene real-time probe was determined by testing serial 10-fold dilutions of RNA extracted from a vaccine strain (LaSota) of NDV with known embryo infectious dose (EID_50_). Samples with a cycle threshold (Ct) value below and including 35 were considered positive for NDV.

**Table 1:** Primers and probes used for M and F gene amplification of Newcastle disease virus.

Targeted gene [Ref.]	Oligo name	Primer/ probe	Primer/probe sequence
**Matrix gene** [12]	M+4100	5’ Primer	5’-AGT GAT GTG CTC GGA CCT TC-3’
	M+4169	Probe	5’-(FAM)TTC TCT AGC AGT GGG ACA GCC TGC-(BHQ]-3’
	M-4220	3’ Primer	5’-CCT GAG GAGAGG CATTTG CTA-3’
**Fusion gene** [14]	VF1	5’ Primer	5’GAY TCY ATC CGY AGG ATA CAA GRG 3’
	V probe	Probe	5’-(FAM)AAR CGT YTC TGY CTC C MGB NFQ 3’
	VR2	3’ Primer	5’AAC CCC AAG AGC TAC ACY RCC 3’

The samples positive for M gene were further tested for fusion gene (F gene) cleavage site by real time RT-PCR using TaqMan MGB probe targeting F gene cleavage site of velogenic/mesogenic NDV strains (Table 1). The sensitivity of the F gene probe for the detection of mesogenic, and velogenic NDV were tested using R_2_B vaccine strain and a genotype VII virus isolate. The lentogenic LaSota strain was used as a negative control. This probe can identify five cleavage site motifs (RRQKRF, RRQRRF, RRRKRF, KRQKRF, GRQKRF), targeting most of the prevalent mesogenic/velogenic strains of NDV [14]. The sensitivity of the F gene assay was found to be 10^2.5^ EID_50_virus. The reaction mixture (12.5μl) contained 2.5μl 5x reaction buffer, 0.4μl forward primer (10pmol), 0.4μl reverse primer (10pmol), 0.3μl probe (5pmol), 0.4μl dNTPs (10mM), 0.5μl enzyme mix, 5μl nuclease free water (NFW) and 3μl template. The volume of RNA was kept constant for RT-PCR reaction irrespective of the initial RNA concentration. The first step was reverse transcription at 50°C for 25 min followed by initial PCR activation for 10 min at 95°C. The next steps included denaturation at 95°C for 10 sec, annealing and extension at 60°C for 30 sec with 45 cycles. The cut off value of 40 Ct was used for F gene.

### Statistical analysis

Apparent prevalence overall of NDV and stratified by risk factors, was calculated along with corresponding 95% confidence intervals (CI) using R statistical software, prop.test function [15]. A series of logistic regression models were run using the glm() function in R. When applicable, true prevalence was calculated using Epitools (https://epitools.ausvet.com.au/trueprevalence) with a Blaker confidence interval based on an estimated RT-PCR assay sensitivity of 90% and specificity of 95% [16,17]. Multivariable logistic regression analysis was conducted using logistic regression in the finalfit function in the finalfit package in R. The outcome was RT-PCR amplification status of M gene (positive vs negative). Independent variables included sex, zone, season (migratory/non-migratory), bird age, migratory bird presence within 3 km of the farm, health condition of bird, bird type, and management system.

## Results

A total of 1371 samples were collected from 157 sites that included 708 samples from backyard chicken, 411 samples from commercial broilers, and 252 samples from commercial layers (Table 2). All commercial layers and broiler chickens flocks were maintained in an intensive management system (n = 40), whereas backyard poultry were raised either in extensive management (n = 60) for owner’s consumption of meat and eggs or in semi-intensive management (n = 57) for commercial purposes. Ten tracheal tissues were also collected from dead birds during study.

**Table 2:** Sampling of chicken according to the management system.

Category	Management system	No. of farms	No of birds sampled
**Backyard**	Extensive/Semi-intensive	117	708
**Commercial broiler**	Intensive	30	411
**Commercial layer**	Intensive	10	252
Total		157	1371

### Prevalence of Newcastle disease virus based on M gene detection

**Fig 2:**
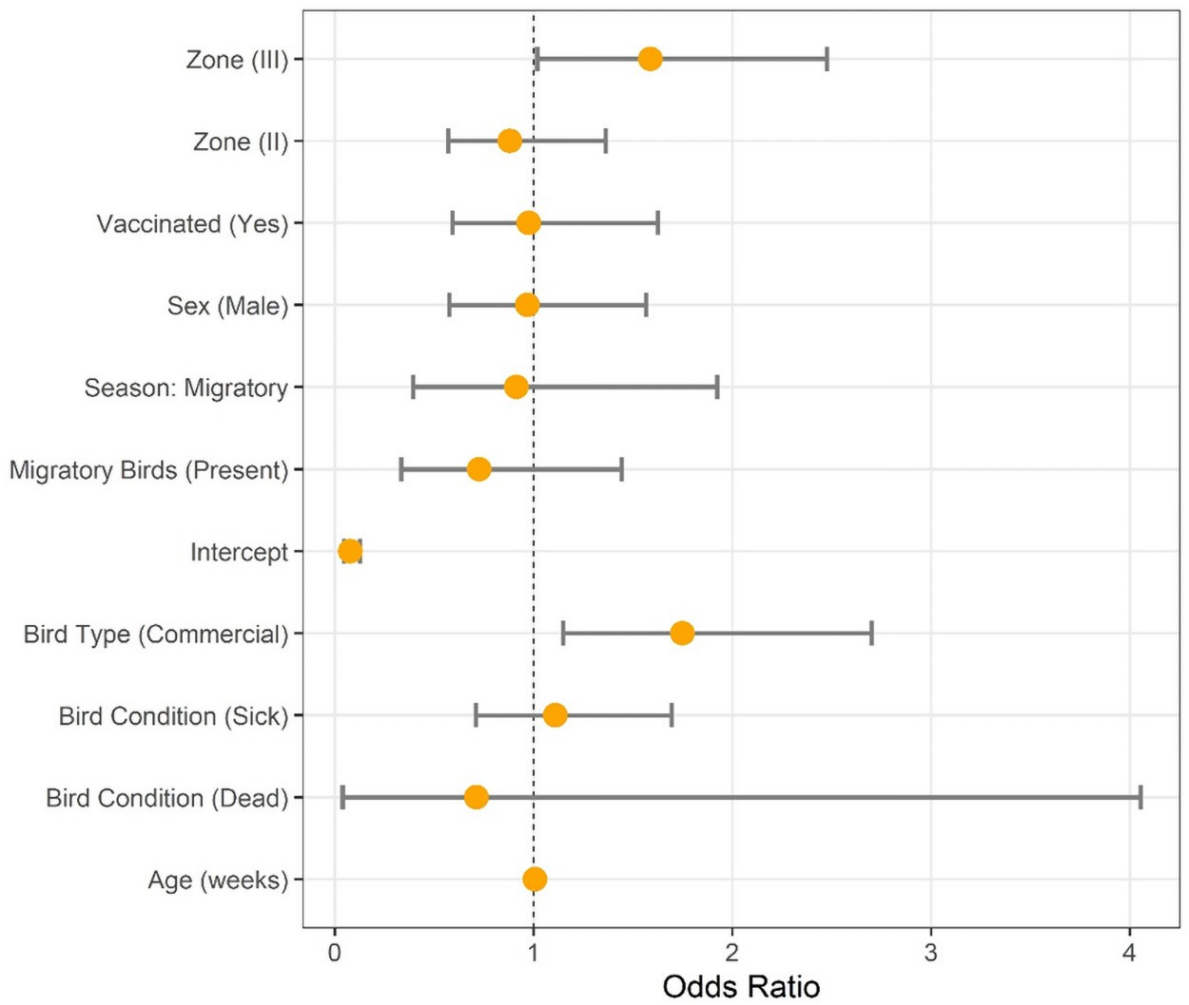
Risk factors impacting Newcastle disease prevalence in 2018 of Odisha state, India.

Overall, 11.7% (95% CI 10.1–13.5) of samples were M gene positive by real-time RT-PCR. The true prevalence based on 90% sensitivity and 95% specificity was 7.9% (95% CI 6.0–10.0). Prevalence of NDV stratified by risk factors such as sex, zone, season, presence of migratory birds, body condition, bird type, housing, management, and bird age is presented in Table 3 and Fig 2. The prevalence was significantly higher among commercial birds (13.7%; 95% CI: 11.2–16.6%) than in backyard birds (9.7%; 95% CI: 7.7–12.2; z = 2.3, p = 0.02). Additionally, NDV prevalence was the highest in zone III (16.6%; 95% CI: 12.7–21.2%) followed by zone I (11.2%; 95% CI: 8.8–14.2%) and zone II (9.1%; 95% CI:6.9–12.1%). The prevalence was not significantly different in female birds (12.1%) than in males (9.5%), non-migratory season (11.7%) than migratory season (11.5%), and areas reporting no migratory birds (11.8%) versus those reporting the presence of migratory birds (10%). Analysis of the data further revealed that there was no significant difference in NDV prevalence among sick birds with respiratory signs (12.7%) as compared to apparently healthy (11.5%) or dead birds (10.0%).

**Table 3:** Odds Ratio with 95% Confidence Intervals of risk factors for prevalence of Newcastle disease virus in Odisha chickens on the basis of M gene detection.

Dependent: status		M gene Negative (%)	M gene Positive (%; 95% C I*)	Multivariable Odds Ratio (95% C I)	P value	Multivariable Risk Ratio (95% CI)
**Sex**	Female	1012 (87.9)	139 (12.1; 10.3–14.1)	-		
	Male	199 (90.5)	21 (9.5; 6.1–14.4)	0.97 (0.57–1.57)	0.900	0.97 (0.61,1.47)
**Zone**	I	482 (88.8)	61 (11.2; 8.8–14.2)	-		
	II	467 (90.9)	47 (9.1; 6.9–12.1)	0.88 (0.57–1.36)	0.568	0.89 (0.6,1.31)
	III	262 (83.4)	52 (16.6; 12.7–21.2)	1.59 (1.02–2.48)	0.041	1.49 (1.02,2.12)
**Season**	Non-migratory	1142 (88.3)	151 (11.7; 10.0–13.6)	-		
	Migratory	69 (88.5)	9 (11.5; 5.7–21.3)	0.91 (0.39–1.92)	0.819	0.92 (0.42,1.74)
**Migratory bird presence**	N	1121 (88.2)	150 (11.8;10.1–13.7)	-		
	Y	90 (90.0)	10 (10.0; 5.2–18.0)	0.73 (0.33–1.44)	0.387	0.75 (0.36,1.37)
**Body condition**	Apparently healthy	982 (88.5)	127 (11.5; 9.7–13.5)	-		
	Dead	9 (90.0)	1 (10.0; 0.5–45.9)	0.71 (0.04–4.05)	0.754	0.74 (0.04,3)
	Sick	220 (87.3)	32 (12.7;9.0–17.6)	1.11 (0.71–1.69)	0.639	1.1 (0.73,1.57)
**Bird type (management)**	Backyard	639 (90.3)	69 (9.7; 7.7–12.2)	-		
	Commercial	572 (86.3)	91 (13.7; 11.2–16.6)	1.75 (1.15–2.70)	0.010	1.63 (1.13,2.32)

*From two-sided proportion test.

A breakdown of NDV prevalence among various bird types by flock size is shown in Table 4. Among extensively managed backyard birds, NDV prevalence was between 10 and 11% (95% CI: 7.2–15.4%) when flock size was less than 200. No positive samples were found in large backyard farms (n = 3) having more than 200 birds. NDV prevalence among commercial broiler chickens increased as flock size increased and was the highest in flocks with >5,000 (21.6%; 95% CI: 14.3–31.3%) birds. In contrast, prevalence among commercial layers was the highest among farms having flock size ≤10,000, which was the smallest flock size reported among intensively reared commercial layers.

**Table 4:** Prevalence of Newcastle disease virus in commercial and backyard poultry based on M gene detection in Odisha.

Bird type	Flock size (Category)	No. of flocks	Total samples collected	M gene positive samples	Apparent Prevalence (95% Confidence interval)	True Prevalence (95% Confidence interval)
**Backyard chickens**	≤20 (S)	50	217	23	10.6 (7.2–15.4)	6.6 (2.5–12.2)
	21–200 (M)	44	429	46	10.7 (8.1–14.0)	6.7 (3.7–10.6)
	>200 (L)	3	62	0	0 (0, 5.8)	0 (0, 1.0)
	Total	97	708	69	9.7 (7.8, 12.2)	5.6 (3.3, 8.4)
**Commercial broilers**	≤1000 (S)	8	83	9	10.8 (5.8, 19.3)	6.9 (1.0, 16.9)
	1001–5000 (M)	17	240	28	11.7 (8.2, 16.3)	7.8 (3.8, 13.4)
	>5000 (L)	5	88	19	21.6 (14.3, 31.3)	19.5 (10.9, 30.9)
	Total	30	411	56	13.6 (10.6, 17.3)	10.2 (6.6–14.5)
**Commercial layers**	≤10000 (S)	2	47	28	59.6 (45.3, 72.7)	64.2 (47.5, 79.3)
	10001–50000 (M)	2	47	3	6.4 (2.2, 17.2)	1.6 (0, 14.3)
	50001–100000 (L)	2	56	4	7.1 (2.8, 17.0)	2.5 (0, 14.1)
	>100000 (VL)	3	102	0	0 (0, 3.6)	0 (0, 0)
	Total	9	252	35	13.9 (10.2, 18.7)	10.5 (6.1–16.1)

S = Small; M = Medium; L = Large; VL = Very Large.

In multivariable analysis, commercial poultry reared under intensive management had 75.0% higher odds of being M gene positive by RT-PCR as compared with backyard poultry under extensive or semi-intensive management (p = 0.01; Odds Ratio [OR]: 1.75; 95% CI: 1.15–2.70). The odds of NDV infection in zone II were 12.0% lower than in zone I (p = 0.568; OR: 0.88; 95% CI: 0.57–1.36) but were 59.0% higher in zone III (p = 0.04; OR: 1.59; 95% CI: 1.02–2.48). The rest of the investigated risk factors did not have a statistically significant difference among levels and the outcome.

### Prevalence of mesogenic/velogenic Newcastle disease virus

Among the 160 M-gene positive samples, 8.12% (13/160; 95% CI: 4.8–13.4%) were positive by the F-gene assay that identifies mesogenic/velogenic strains. Out of 13 psotitive samples, 02 samples were sick and the remaining 11 samples were apparently healthy. The samples positive for F gene included commercial broiler chickens (n = 6) and backyard poultry (n = 7). Only one sample from each category was sick and all others were apparently healthy. None of the samples from commercial layer was F gene positive. This indicates the circulation of potentially virulent NDV strains amongst healthy poultry population in Odisha (Table 5). The true prevalence of mesogenic/velogenic strains was 3.7% (95% CI: 0–9.9%).

**Table 5:** Detection of NDV in various types of birds.

Zone	Bird Type	No. of samples positive for M gene	No. positive for F gene
I	Backyard	38	6
	Commercial broilers	16	1
	Commercial layers	7	0
II	Backyard	11	0
	Commercial broilers	32	4
	Commercial layers	4	0
III	Backyard	20	1
	Commercial broilers	8	1
	Commercial layers	24	0

## Discussion

The present study was undertaken to determine the prevalence of NDV in apparently healthy commercial poultry and backyard birds in the state of Odisha, India. Most of the earlier studies on ND in India have been undertaken in outbreak situations where the disease was detected and virus was isolated and characterized [18,19]. In contrast, this study was undertaken in apparently healthy flocks in Odisha state. The aim of the study was to understand the nature of circulating NDV in the poultry population of the state. We believe that such studies in apparently healthy birds would provide data on the presence or absence of the virus in these flocks, which can then be used to formulate appropriate control programs.

We adopted a two-step NDV detection process; in step 1, we tested by M gene probe to detect the presence of NDV and in step 2, we tested M gene positive samples by an F gene probe to detect mesogenic/velogenic NDV. The results indicate circulation of lentogenic and mesogenic/velogenic strains in commercial broilers and backyard poultry. Circulation of lentogenic NDV in commercial broiler, commercial layer, and backyard flocks has been reported in Ethiopia [20]. Although lentogenic strains cause a milder form of the disease, their presence is still a concern because of their role in virus evolution. Thus, it has been reported that lentogenic NDVs with passage may become virulent and can cause disease [21].

Multivariable logistic regression based on M gene results in our study revealed that zone III had significantly higher prevalence as compared to zone I and that commercial poultry had significantly higher odds of being infected with NDV compared to backyard poultry. Zone III is mostly dominated by tribal population with a lower rate of literacy as compared to that in zones I and II [22]. Furthermore, a high density of backyard poultry with co-rearing of birds of different age groups in zone III, as recorded during the study period, could also contribute to higher NDV prevalence observed in this region. Co-rearing (rearing of backyard poultry, wild birds including pigeons, and commercial birds in the same farm) can lead to disease transmission among various bird types. Moreover, extreme climatic conditions of rain and temperature in zone III might be acting as added stressors [23]. These observations may help in developing and implementing region-specific disease containment and biosecurity plans. We did not find a difference in NDV prevalence between migratory (winter) and non-migratory seasons. This is in contrast to Nwanta et al. who reported higher occurrence of NDV in dry (winter) season in Nigeria [24]. Resolving this issue is challenging, as the current practice of maintaining commercial birds in intensive systems and indigenous birds in semi-intensive or extensive systems of rearing may confound results. Further epidemiological studies are required on this aspect.

Of the M gene positive samples, 8.12% were positive for the F gene indicating the presence of mesogenic/velogenic strains in backyard and commercial broiler chickens. In commercial broiler farms vaccination with F1/ lentogenic strains of NDV is done invariably in the first week of age. It is believed that vaccination against NDV helps protect the birds against mortality and clinical disease but may not stop viral shedding, which may provide an opportunity for circulation of NDV in a commercial flock or at commercial-backyard interface. Therefore, circulation of mesogenic/velogenic NDV in commercial broilers underlines the importance of NDV surveillance in healthy flocks. The mesogenic strains (R_2_B) of NDV are used for vaccination in commercial layer birds; however, none of the samples of commercial layers was positive for mesogenic/velogenic NDV strains in this study. Previous studies have reported on the presence of velogenic NDV in healthy birds [20,25] as was seen in this study by F gene PCR confirmation of mesogenic/velogenic NDV in healthy commercial broilers and backyard poultry.

Backyard poultry farmers seldom practice vaccination in this region. The role of backyard poultry in the epidemiology of NDV is a big concern in countries where backyard poultry rearing is common. Virulent strains of NDV have been reported from backyard poultry [26–29]. Thus, NDV positive apparently healthy backyard flocks have a great potential to transmit velogenic NDVs to commercial poultry. Further, error prone genome transcription during replication of RNA viruses such as NDV helps viruses escape host immune defense and evade diagnostic tests [26]. In this study, we tested samples for the detection of NDVs only. Studies are underway to perform whole genome sequencing of these samples to ascertain the genotypes of NDVs circulating in these F-gene positive flocks.

The results of this study highlight the importance of NDV surveillance in healthy populations of birds and provide baseline information on circulation of mesogenic/velogenic NDV in healthy birds. The findings suggest that mesogenic/velogenic NDV strains are circulating and are perhaps endemic within poultry population of Odisha. In this study, we only used F gene amplification to discriminate between lentogenic and mesogenic/velogenic NDV strains. We did not isolate and characterize the virus. Even lentogenic strains can often cause disease. For example, Nagy et al. (2020) found that 2 of 13 NDV isolates whose F-gene cleavage site motif indicated them to be lentogenic strains, but were found to have high intracerebral pathogenicity index and mean death time suggesting them to be virulent [30]. The data generated in this study point out the need for further work with maximize sample collection across the state with risk factor stratification, and in-depth analysis of viral genotypes.

## Conclusions

This study aimed to fill the knowledge gap of Newcastle disease in Odisha, an eastern state of India, by investigating NDV prevalence based on the detection of M and F genes. The surveillance study revealed an overall apparent prevalence of NDV of 11.7% among the commercial poultry and backyard birds of Odisha along with the circulation of lentogenic as well as mesogenic/velogenic NDV strains. These results provide baseline information on the endemic nature of NDV in the state. The mesogenic/velogenic NDV circulation in healthy birds and the geographically close association between commercial and backyard poultry in the region underline the need for cross-sectional studies on commercial and backyard poultry.

## Supporting information

S1 Table Information on ND vaccination status, respiratory signs, location and real-time PCR results of the samples collected from domestic chicken of Odisha, India. (XLSX)
